# A Re-Appraisal of the Early Andean Human Remains from Lauricocha in Peru

**DOI:** 10.1371/journal.pone.0127141

**Published:** 2015-06-10

**Authors:** Lars Fehren-Schmitz, Bastien Llamas, Susanne Lindauer, Elsa Tomasto-Cagigao, Susan Kuzminsky, Nadin Rohland, Fabrício R. Santos, Peter Kaulicke, Guido Valverde, Stephen M. Richards, Susanne Nordenfelt, Verena Seidenberg, Swapan Mallick, Alan Cooper, David Reich, Wolfgang Haak

**Affiliations:** 1 Department of Anthropology, University of California Santa Cruz, Santa Cruz, California, United States of America; 2 Historical Anthropology and Human Ecology, University Goettingen, Goettingen, Lower Saxony, Germany; 3 Australian Centre for Ancient DNA, School of Biological Sciences, The University of Adelaide, South Australia, Australia; 4 Curt-Engelhorn-Center for Archaeometry, Mannheim, Baden-Württemberg, Germany; 5 Departamento de Humanidades, Pontificia Universidad Católica del Perú, Lima, Perú; 6 Instituto de Investigaciones Arqueológicas y Museo, Universidad Católica del Norte, San Pedro de Atacama, Antofagasta, Chile; 7 Department of Genetics, Harvard Medical School, Boston, Massachusetts, United States of America; 8 Departamento de Biologia Geral, Instituto de Ciências Biológicas, Universidade Federal de Minas Gerais, Belo Horizonte, Minas Gerais, Brazil; 9 Broad Institute of Harvard and MIT, Cambridge, Massachusetts, United States of America; 10 Howard Hughes Medical Institute, Boston, Massachusetts, United States of America; University of Oxford, UNITED KINGDOM

## Abstract

The discovery of human remains from the Lauricocha cave in the Central Andean highlands in the 1960’s provided the first direct evidence for human presence in the high altitude Andes. The skeletons found at this site were ascribed to the Early to Middle Holocene and represented the oldest known population of Western South America, and thus were used in several studies addressing the early population history of the continent. However, later excavations at Lauricocha led to doubts regarding the antiquity of the site. Here, we provide new dating, craniometric, and genetic evidence for this iconic site. We obtained new radiocarbon dates, generated complete mitochondrial genomes and nuclear SNP data from five individuals, and re-analyzed the human remains of Lauricocha to revise the initial morphological and craniometric analysis conducted in the 1960’s. We show that Lauricocha was indeed occupied in the Early to Middle Holocene but the temporal spread of dates we obtained from the human remains show that they do not qualify as a single contemporaneous population. However, the genetic results from five of the individuals fall within the spectrum of genetic diversity observed in pre-Columbian and modern Native Central American populations.

## Introduction

**Fig 1:**
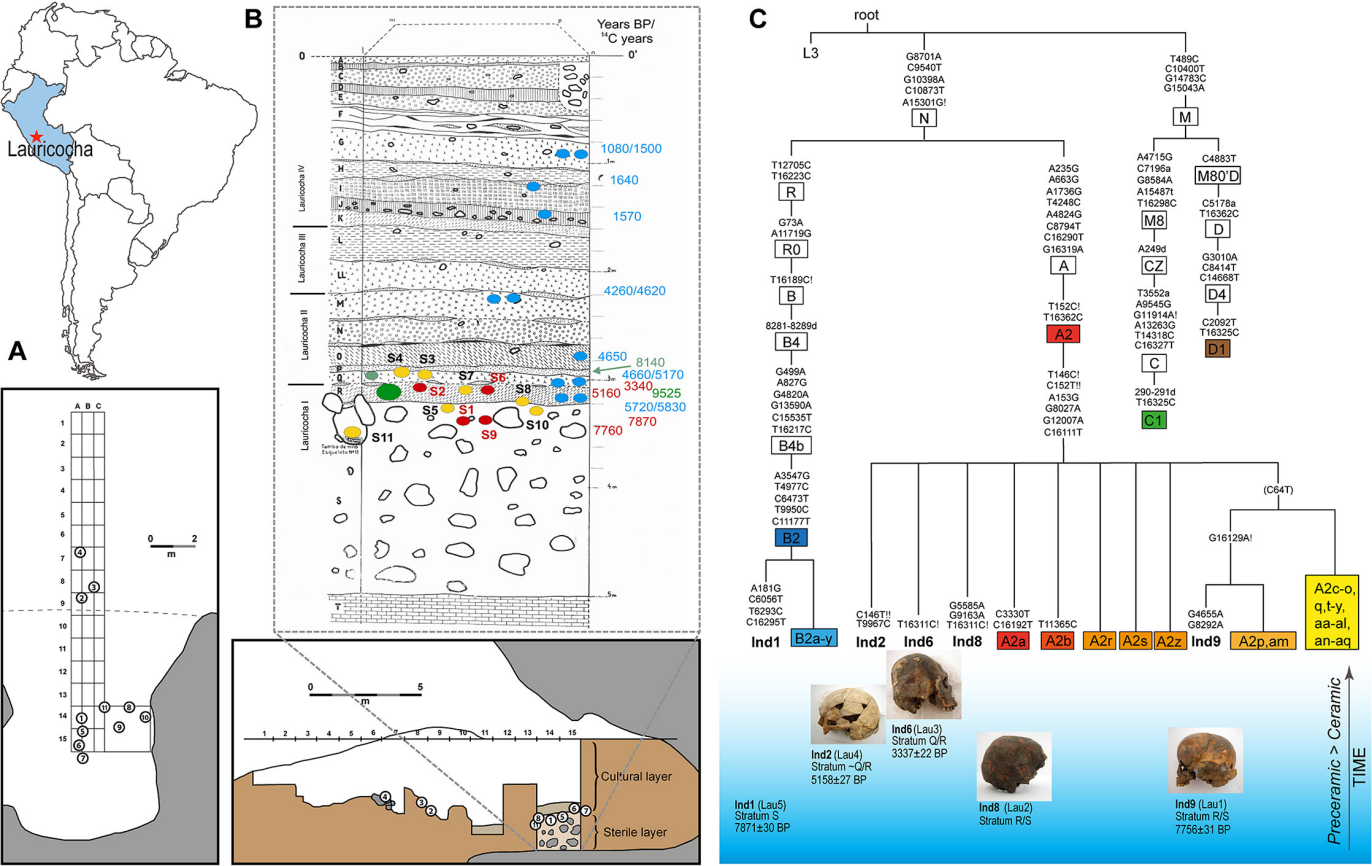
Geographic, stratigraphic, and genetic data for the Lauricocha Cave. (A) Geographic position of Lauricocha in South America. (B) Stratigraphy of the Lauricocha Cave in combination with the radiocarbon dates obtained in this (red) and previous studies (Groningen/blue and Teledyne/green) and the positioning of the human remains within the archaeological site (numbers in circles correspond to individual numbers) (C) Phylogenetic placement of the mitochondrial genomes obtained from the Lauricocha individuals in the diversity of the Native American haplogroups A2 and B2 and thumbnail images of the skulls and their chronology (bottom right).

The peopling of the high altitude Central Andes marks an important episode in South American population history, eventually leading to the formation of the most complex societies of the late pre-Columbian period, namely Wari, Tiwanaku, and Inca. Archaeological evidence indicates human presence along the Central Andean coastline around 13,000 to 14,000 years ago. [1,2]. Despite the harsh conditions and physical stressors, humans started almost immediately to live seasonally at high altitude (more than 2,500 meters above sea level) in the Central Andes, and permanent residence becomes evident about 12,000 to 10,000 years ago [3–5]. The first sites occupied by Andean high altitude foragers were caves and rock shelters [3,6–8]. Such rock shelters are found at Lauricocha in Peru, located at ~4050 meters above sea level near Lake Lauricocha and the source of the Marañón River (Fig 1). Augusto Cardich excavated the site in several campaigns between 1958 and the early 1960’s and found incomplete skeletal remains of 11 humans (8 adults, 3 sub-adults) along with lithic tools and burnt and unburnt animal bones in the lower layers of the site stratigraphy. Cardich initially obtained two radiocarbon dates from these lower layers from Teledyne Isotopes (USA), suggesting that all burials were older than ~8100 uncalibrated radiocarbon years (^14^Cyr). The Lauricocha site soon gained iconic status as it showcased the first evidence for an Early Holocene presence of humans in the high Andes [8].

Anthropological examinations of the skeletons from Lauricocha were performed on three of the best-preserved skulls (individuals 2, 6, and 8) by Marcello Bormida in the early 1960’s [9]. The study showed that the Early Holocene skulls 2 and 8 were dolichocephalic (long and narrow). This specific form of the skull vault often has been described in older literature as a morphological trait of Paleoamericans, the Late Pleistocene and Early Holocene inhabitants of the Americas [10,11]. In contrast, mesocephalic (average or medium cranial vault) and brachycephalic cranial shapes (short and wide cranial vault) are often described as characteristic of more recent Native American populations, and have been interpreted as evidence for the introduction of further biological diversity into the continent during the later Holocene [11,12]. The skull vault form of the two dolichocephalic individuals thus was seen as consistent with the asserted Palaeoamerican ancestry of the Lauricocha individuals. Consequently, many subsequent studies used the individuals from Lauricocha as morphological representatives of Early Holocene/Middle Pre-ceramic Paleoamerican populations of the Central Andes [10,11]. Another observation from Bormida was the artificial occipital flattening of skull 6, thought to be from the Early Holocene, and therefore considered to be the oldest case of artificial cranial modification in South America [8,9]. However, younger radiocarbon dates from later excavations at Lauricocha analysed in Groningen in the 1960’s cast doubts on the antiquity of the site, and raised questions with regards to the relevance of the Lauricocha human remains for the understanding of Andean population history and the initial peopling of South America [6,13,14]. The new results dated the lower layers of the site ~3000 ^14^Cyr younger. Additional early high altitude sites have been identified after the discovery of Lauricocha, such as Cuncaicha, Guitarrero Cave, Pachamachay and Telarmachay [3,4,6]. While these recent discoveries are better documented, Lauricocha nonetheless represents one of the few early sites with a larger number of human remains.

Here we re-evaluated the dating of the human remains found at Lauricocha by generating new radiocarbon dates directly from skeletal elements and contextualizing these dates with the dates from the Teledyne and Groningen labs and stratigraphic information. We also re-assessed the initial morphological and morphometric investigations, and generated complete mitochondrial genomes and nuclear genetic data to genetically characterize the Lauricocha individuals. Our investigations not only support the Early/Mid-Holocene age of Lauricocha but also suggest genetic continuity in the region throughout Andean prehistory.

## Materials and Methods

### Site and samples

Lauricocha Cave L-2 is a rock shelter of ~15m depth situated in a cliff and part of a complex consisting of three shelters/caves [8]. The cave complex is located at ~4050 meters above sea level, overseeing the Lauricocha Lake and close to the source of the Marañón River. The closest modern settlement is Lauricocha, province of Lauricocha, department of Huanuco, on the opposite side of the lake. The site was discovered by Augusto Cardich in 1957, and a series of archaeological excavations followed until 1968 [8,13,15–17]. The main excavation of Cave L-2 consisted of a 15m trench from the entrance of the cave to the back with a maximum depth of 5m, at which the bedrock of the cave was located. Above the bedrock, Cardich found a ~1.7m-high sterile glacio-fluvial layer (Layer S) that likely formed the cave floor the first inhabitants encountered. Above Layer S there are ~ 3.3m of sediment layers containing evidence for human presence, *e*.*g*. lithic tools, ceramics, hearths, and animal bones ranging from the Early Pre-ceramic Period to the Inca Period. Cardich’s stratigraphy lists 20 distinct layers in total (Fig 1).

The human remains found in the cave were found in two distinct areas. Individuals 2, 3, and 4 were found at the entrance to the rock shelter at a depth of 2.7–3.15m. All other individuals were found at the back of the rock shelter between the lowest three layers S, R and Q (Table 1). These lower layers are characterized by a relative high concentration of calcite and an alkaline soil (pH 8.6) [8]. All adult individuals were buried in flexed, lying position. While the individuals found in the lowest layer S were buried in shallow grave pits, no information on the grave construction for the individuals in the upper layers is available. The adult graves contained small amounts of grave goods, *i*.*e*. one or two lithic tools and mammal bones. The graves of the subadult individuals were richer, including pieces of ocher, stone and bone tools, calcinated mammal bones, food and pearls. For example, red ocher was found next to individual 9, yellow ocher in grave 10, and individual 11 was covered in grains of iron oxide.

**Table 1:** Summary of context information for all Lauricocha skeletal remains.

Skeleton	Depth (m)	Square	Layer	Sex	Age (yrs)	Samples	Lab code	^14^C yrs BP (uncal)	Calibrated dates (1 sigma)	mtDNA HG	Y-Chr HG
1	3.3–3.4	14A,14B	S	Female	Adult—Mature	S07: Molar 1/7				B2	
						S08: Metatarsal, 1st right	MAMS 14731	7871 ± 30	6641–6534 cal BCE	B2	
2	3.15	9A, 9B	Q, R	Male	30–50	S05: Molar 3/8				A2	Q1a3a*
						S06:Metacarpal, 3rd right	MAMS 14390	5158 ± 27	3988–3955 cal BCE	A2	Q1a3a*
3	2.85	8B, 8C	Q								
4	2.7	7A, 7B	Q								
5	3.2–3.3	14A, B, 15A,15B	S								
6	3–3.2	15A,15B	Q, R	Male	60	S03: Molar 3/8				A2	Q1a3*
						S04: Metatarsal, 2nd left	MAMS 14389	3337 ± 22	1682–1611 cal BCE	A2	Q1a3*
7	3–3.2	South of 15A	Q, R		Juvenile						
8	3.15–3.3	E	R, S	Male	50+	S02: Pars petrosa				A2	n.d.
9	3.4–3.6	E	S	Male	1.5–2	S01: Pars petrosa	MAMS 14391	7756 ± 31	6750–6648 cal BCE	A2	Q1a3*
10	3.25–3.4	E	S	Unknown	12						
11	3.4–3.6	13C, 14C, E	S	Male	2						

We include archaeological coordinates, anthropological sex and age at death, type of sample, uncalibrated radiocarbon dates (uncal) and calibrated dates (cal), and mitochondrial and Y-chromosomal haplogroups (HG).

The remains of individuals 1, 2, 6, 8, and 9 (specimen numbers: Lauricocha Esqueleto 1, 2, 6, 8, 9) are currently held at the Museo Nacional de Arqueología, Antropología e Historia del Perú (MNAAHP) in Lima, Peru. The permission for research, destructive analysis and exportation was granted to LFS and ETC by the Instituto Nacional de Cultura del Perú (Permit Number: Resolucion Directoral Nacional 1346), Lima, Peru. The whereabouts of the remaining skeletons is unknown to us. During our field campaign in 2010 we were granted access to the specimens at the MNAAHP to perform the morphological and craniometric re-analysis, to make 3D scans of the skulls, and to take bone and tooth samples for AMS radiocarbon dating and ancient DNA analyses. We acquired eight samples from the five individuals listed in Table 1.

### AMS radiocarbon dating

Bone samples from individuals 1, 2, 6, and 9 were sent to the Curt-Engelhorn-Center for Archaeometry—MAMS for AMS radiocarbon dating, using 500mg of each bone sample for collagen extraction. For individual 8 it was impossible to sample enough material for both radiocarbon and paleogenetic analyses, so we decided to only perform the genetic analyses.

Bone decalcification was performed using several acid steps (4% HCl) at room temperature on the first day, followed by a base step (0,4% NaOH) and another subsequent acid step on the second day. To extract the collagen the bone material was gelatinized at pH 3 (HCl) at 60°C for 20 hours. Solid components were separated using Ezee-filter separators (Elkay, UK) and molecules smaller than 30kDa were separated via Ultrafiltration. Isolated collagen was frozen for 2 days and then freeze-dried. Samples were graphitized (converted to gaseous CO2) using an elemental analyzer (Elementar), cryogenically collected, and transferred to a reactor [18]. Iron was used as a catalyst to convert CO_2_ with addition of H_2_ to H_2_O and elemental C, while H_2_O was reduced by freezing. The Fe-C mixture was than pressed into a target and measured with the MICADAS System [18,19]. The use of the elemental analyzer also allowed us to determine the carbon/nitrogen (C/N) ratio which is a criterion for the quality of the collagen [18]. C/N ratios of 3.4 to 3.5 were determined in the tested samples proving a sufficient collagen quality. Radiocarbon dates and C/N ratios are given in S1 Table.

To detect contamination during sample preparation, two modern bone samples (internal laboratory standards, one calf, 6 month old, from the butcher, one pig bone buried in a garden of known age and burial time) were prepared together with the skeletons. The same consumables were also used on a mammoth bone sample (Latton Mammoth). The age of the mammoth bone exceeds the dating limits of the applied radiocarbon dating methods. Thus, any detectable age would have indicated modern contamination due to handling processes or consumables.

The new radiocarbon ages from this study as well as the dates obtained at Teledyne and Groningen were calibrated using OxCal v4.2 together with the IntCal13 dataset [20,21]. We did not apply an age correction for the southern hemisphere because Lauricocha lies north of 10°S, and hence is not supposed to be influenced by shifts of the ITCZ (Inter-Tropical-Convergence-Zone) and its resulting climatic changes [22,23]. Calibrated and uncalibrated dates of all available Lauricocha radiocarbon samples are given in S1 Table and S1 Fig.

### Morphological and craniometric analyses

Our team was the first to reanalyze the human remains of Lauricocha after the initial examinations by Bormida [8,9]. For each individual we performed an overall assessment of the degree of skeletal integrity, noting the missing bones or parts of bones, and comparing them to the original publication. In addition, the degree of fragmentation, fragility, and taphonomic changes were assessed. Methods employed to determine key parameters as sex, age at death, stature, as well as anatomical variants, and pathological, traumatic and habitual alterations are described in S1 Materials and Methods.

The cranial measurements were recorded from high-resolution 3D digital models made by a NextEngine HD laser surface scanner and ScanStudio software interface (Version 1.3.2). The completed 3D models were imported into Stratovan checkpoint digitizing software (Version 2014.07.21.0322). Measurements of cranial length and breadth were recorded in millimeters using the landmark selection and length primitive tools. The purpose of the cranial index is to determine if a dry skull falls within one of four ranges, which are described by Bass [24] as follows: dolichocrany (narrow or long headed), mesocrany (average or medium), brachycrany (broad or round), or hyperbrachycrany (very broad). To obtain the indices, cranial measurements were recorded following the measurement descriptions and formula from Bass [24] and White et al. [25]. Cranial breadth was measured from the right and left *Euryon* landmarks, the two most widely separated points on each side of the skull. Cranial length was measured from *Glabella* (the most anterior point of the skull at the midline) to *Opisthocranion* (the most posterior point of the skull). Further details can be found in SI Material and Methods.

### Paleogenetic analyses

The eight samples from the five individuals from Lauricocha were collected at the MNAAHP taking all possible precautions through the use of gloves, face masks, protective clothing and decontamination of the working surface and tools with 3% commercial bleach. We collected two samples each for individuals 1, 2, and 6: one molar, and one metatarsal or—carpal. We sampled the complete *pars petrosa* of the temporal bone for individuals 8 and 9. Teeth were extracted from their alveolar sockets, and petrosal bone was cut using a Dremel drill with diamond disks. The samples were initially shipped to the ancient DNA (aDNA) facilities at the Department of Anthropology, University Goettingen, Germany (GoA) where they were stored at -20°C. Hereafter, genetic investigations (*e*.*g*. DNA extractions, PCR, genomic library preparation, hybridization capture) were conducted independently at three additional institutions: the Australian Centre for Ancient DNA, University of Adelaide, Australia (ACAD), the Reich Lab at the Department of Genetics, Harvard Medical School, USA (HMS), and the Human Paleogenomics Lab at the Department of Anthropology, UCSC, Santa Cruz, USA (UC-HPL). Bone powder samples were initially distributed to these labs from GoA. Detailed descriptions of contamination prevention procedures, sample preparation, and DNA extraction are described in S1 Material and Methods. Nuclear genetic data in this study was obtained via PCR-based experiments and analyzed employing capillary electrophoresis (protocols and primer sequences, S1 Material and Methods) at GoA and UC-HPL. To assess the authenticity of PCR- based results, we performed at least four independent amplifications from two independent DNA extracts each for every genetic marker, resulting in a minimum of 8 amplification results. We used a majority call to determine a consensus for each allele. Complete mitochondrial genomes were obtained at ACAD, HMS, and UC-HPL via targeted enrichment from double barcoded sequencing libraries, using hybridization capture assays, followed by high-throughput sequencing on Illumina Next Generation Sequencing platforms. Refer to S1 Material and Methods for details regarding applied protocols and downstream analysis of the sequencing data.

## Results and Discussion

### Chronological context of the Lauricocha skeletons

Cardich’s stratigraphy partitioned the burials of Lauricocha into two phases (Fig 1). Individuals 1, 5, 9, 10 and 11, found in small shallow grave pits in the lowermost sterile glazifluvial layer S, represent the oldest phase, whereas individuals 2, 3, 4, 6 and 7, found in the overlying cultural layers R and Q, are part of the younger phase (Fig 1, Table 1). Individual 8, buried between layers R and S, is intermediate. Two radiocarbon dates (Teledyne Isotopes, USA; green dates in Fig 1B) were obtained by Cardich from a mixture of burned and unburned bone and sediments to estimate the age of the burials. The oldest date (9525 ± 260 ^14^Cyr) from layer R and the other date (8140 ± 140 ^14^Cyr) from layer Q suggested that all burials, located below layer Q, were older than ~8100 ^14^Cyr. However, additional radiocarbon dates (Groningen, Netherlands) from sediment and ash samples throughout the whole stratigraphy commissioned during later excavations at Lauricocha were not consistent with the earliest claims [13]. The Groningen dates obtained for layers R and Q (5830 +/- 120 and 4660 +/- 90 ^14^Cyr, respectively) were significantly younger than the Teledyne dates (Fig 1B, blue dates). To resolve the chronology of human presence in Lauricocha, we directly dated bones from individuals 1, 2, 6, and 9 (curated at the Museo Nacional de Arqueología, Antropología e Historia del Perú) using accelerator mass spectrometry (AMS) radiocarbon dating (S1 Fig). The good preservation status of the bones from Lauricocha allowed the extraction of sufficient amounts of collagen from the samples. Individual 1, a mature woman, and individual 9, an 2–4 year-old infant, both from layer S at the back of the cave, were dated to 7871 ± 30 ^14^Cyr and 7756 ± 31 ^14^Cyr, respectively. These remains are stratigraphically associated with Individuals 5, 8, 10 and 11. Results were surprisingly heterogeneous for the interface between layers Q and R: individual 2, a young adult male buried at the front of the cave, was dated to 5158 ± 27 ^14^Cyr and matches the Groningen dates from layers above and below. However, individual 6, a ~40 year old man buried at the back of the cave, was dated to 3337 ± 22 ^14^Cyr (cf. Table 1). Interestingly, both older Teledyne Isotopes dates neither correlate with Groningen dates from the sediment samples from the same layers [13] nor with the newly obtained radiocarbon dates directly from the skeletons (this study) (Fig 1). The bones dated by us clearly exhibit younger ages for Layers S, R, and Q than those estimated from the Teledyne data (S1 Table). However, our dates are consistent with the Groningen dates of the stratigraphy when taking into account the general intrusive character of burials. Given the available information on layer designation, depth, and sediment structure, the Groningen radiocarbon dates support our date for individual 2 and our older ages in Layer S (S1 Table and Fig 1). However, our date for individual 6 correlates with the much younger layers L to LL. The difference in depth of ~1m between layer L and the position of individual 6 (between layers Q and R) suggests that this interment cuts through older cultural deposits. The excavation techniques used by Cardich would have not allowed him to identify the intrusive character of this burial [8].

Cardich reported that a mixture of burnt and unburnt bones and small fragments of charcoal were used for dating at Teledyne Isotopes [8]. These two samples were leached in acid to eliminate inorganic carbon. Today we know that burnt bone consists mainly of bone apatite which is known to exchange carbon with the surrounding soil [26,27], but sample pre-treatments that allow dating of the bio-apatite fraction in burnt or cremated bone to overcome the contamination effect on radiocarbon results had not yet been available in the 1960’s [26]. Indeed, the soil of the Lauricocha Cave site has been reported as being rich in carbonate [17]. The age of soil carbonate is usually geologic and thus would represent a contamination leading to an older date. Referring to the information that the original sample consisted of a mixture with organic material it is possible that the organic material in question might have been charcoal. Charcoal might suffer from the so-called old-wood effect. This refers to the fact that charcoal is burnt wood, which usually grows in rings. If we now sample a ring from the inner (older) part, the date we receive from radiocarbon dating can be significantly older than the event to be dated [26]. However, in the case of Lauricocha it has to be expected that enough wood was available in the ecosystem and the inhabitants did not have to rely on gathering older dead wood. Thus it is unlikely that the old-wood effect is a considerable factor. Therefore, we propose that the much older ages were likely derived from residual carbonate contaminations from the cave. Overall, our refined dating results support three discrete burial phases (two during the Pre-ceramic Period, 9000–1800 cal BCE, one during the Formative Period, 1800–800 cal BCE), suggesting the number of burial phases had been underestimated. More importantly, our results confirm the initially proposed early occupation of the Lauricocha site almost 8,000 years ago, which had been challenged previously [6], and thus restore Lauricocha’s significance as one of the oldest highland sites with human remains in South America.

### Morphological analysis of the human remains

Our re-assessment of the age of the Lauricocha human remains provides an opportunity to contrast both the cranial morphology and the mitochondrial genetic diversity of Andean individuals who lived before and after the Middle Holocene. We performed a new series of morphological and craniometric analyses on individuals 1, 2, 6 and 8 (S2 Fig), and confirmed Bormida’s observations that the incomplete skull of ~8,550-year-old individual 8 is indeed dolicocephalic (cranial breadth 131.4 mm, length 194.6, cranial index 67.52 = dolicocrany) [9]. However, we show that the skull of ~5900-year-old individual 2 is mesocephalic (cranial breadth 135.2 mm, length 178, cranial index 75.96 = mesocrany, *i*.*e*. average or medium cranial vault), while the partial skull of individual 6 is inconclusive (S3 and S4 Figs). We also found that the latter skull does not date to the Early Holocene but to the Late Holocene (~3337 ± 22 ^14^Cyr BP), and cannot be definitively classified as an artificially deformed skull. The younger date coincides with the wider spread of this cultural practice during the Late Archaic and Formative Period (~3,000–800 cal BCE) in the Central Andes [28–31]. Even though radiocarbon dates show that individuals 1 and 8, 2, and 6 are not contemporaneous, they share several morphological characteristics. Dental health is similar, with severely worn teeth showing flat and oblique occlusal surfaces, hypercementosis and peri-apical abscesses, and few, shallow, interproximal cavities. The attachments of the masticatory muscles are very strong and there are slight to moderate degenerative changes in the temporo-mandibular joints. These shared characteristics indicate that a mixed diet containing mainly tough, unprocessed foods and few carbohydrates [32–34] remained almost unchanged through time. The severe dental attrition prevented the recording of linear enamel hypoplasia excepting in skeletons 2 and 6 that preserved at least a part of the crowns. Individual 2 showed one line, and individual 6 showed two. According to the developmental charts of Reid and Dean [35], these lines possibly formed around 5 years in the first case and between 2.5 and 4 years in the second.

The postcrania of individuals 1, 2 and 6 are very robust, with strong muscular attachments visible in arms and legs. This suggests there were also no significant changes in the intensity of activities through time. In skeleton 1 the degenerative changes of the cervical column are much more pronounced than those recorded in the lumbar region, suggesting activities that affected the neck. This individual also showed advanced degenerative changes in the head of the right radius and the 2nd right intermetatarsal joint, the only bones preserved from elbow and foot. Intermetatarsal arthritis is not frequent, which means that the severe changes in this individual might be due to activities that overstressed the feet. Skeleton 2 shows erosion in the posterior surfaces of the femoral condyles, as well as tibial imprints [36] and strong insertions for the *vastus lateralis* in the left patella (right patella not preserved). These indicators suggest that individual 2 spent substantial amounts of time in a squatting position.

Skeletons 8 and 2 show evidences of severe infection. The maxilla of individual 8 has a very strong, active bone reaction that extends through the palatine surface and the floor of the right nasal cavity. Since this is a localized lesion, it was possibly transmitted from the teeth. In contrast, infections diagnosed in skeleton 2 appear systemic and chronic, since the reaction occurred in areas of muscular attachment at elbow and knee and showed both active and remodeled areas. This skeleton also showed slight spongiosclerosis in the cranial vault and in the roof of the left orbit, indicating anemia. Finally, skeleton 6 showed healed, unspecific periostitis in the left clavicle.

### Paleogenetic analysis of the human remains

We extracted the DNA from tooth and bone samples for the five individuals 1, 2, 6, 8, and 9 to perform genetic analyses. After an initial screening of the mitochondrial D-loop using PCR and Sanger sequencing, we could assign individuals 1, 2, 6, 8 to mitochondrial haplogroup A2, and individual 9 to B2 (Table 1, S3 Table). Both haplogroups are founder mitochondrial lineages in the Americas [37]. We then performed hybridization capture of the mitochondrial genome for all individuals independently in the Boston and Adelaide laboratories, producing consistent results. Complete high coverage mitochondrial genomes (average coverage depth ranging from 43.7–106.2x) were obtained for individuals 1, 2, 6, and 9 confirming and refining initial haplogroup calls made via PCR (S4 Table). We also obtained a complete mitochondrial genome with lower coverage for individual 8 (average coverage depth of 14x), confirming the haplogroup A2 assignment (see S4 Table for all sequencing statistics). All mitochondrial genome sequences are available from GenBank (accession numbers: KP300790-KP300794). The high frequency of mitochondrial A2 haplotypes in Lauricocha (4 out of 5 individuals) is intriguing because modern-day Central Andean populations exhibit high frequencies of haplogroup B and only minor frequencies of A. However, previous ancient DNA studies also found a much higher frequency of haplogroup A in Pre-ceramic Central Andean populations [38,39]. Phylogenetic comparison reveals that the ancient A2 mtDNA genomes have not been described previously and form discrete sister-clades basal and/or within the diversity of modern South American populations (Fig 1C). This is also true for the B2 haplotype of the Early Holocene individual 1, which is novel and phylogenetically nested within the diversity of this Native American founding lineage. The five sequence haplotypes show no more than between 1 and 4 mutations difference from their respective proposed founder lineages (i.e., basal A2 and B2), which falls well inside the mutational spectrum of modern-day Native American A2 and B2 lineages (PhyloTree.org—mtDNA tree Build 16 (19 Feb 2014)). We would need more data from ancient and modern-day Andeans at the same level of genetic resolution to formally test for population continuity or discontinuity at a regional scale, as has been shown for samples from North America [40]. In fact, a recent HVR-I based study from the Central Andes reported population movements and exchange that impacted on the genetic structure of the Central Andean gene pool [41], suggesting episodes of population discontinuity. At present, the new results from Lauricocha can only be interpreted at a broad scale, where the five individuals likely trace their ancestry back to a founding population that led to all Native American populations in the region [42].

To examine the evolutionary history further, we also genotyped several chromosomal markers. We determined the Y-chromosome haplogroup of male individuals 1 and 6 to be Q1a3a* (xM3), and Q1a3a1* (Q-M3) for individual 2. Both haplogroups are Y-chromosome founder lineages in the Americas diverging approximately 16,900 years ago in Beringia [43]. They are found in contemporary Andean populations, with Q1a3a1* being the dominant haplogroup and Q1a3a* only occurring at low frequency [44,45]. Network analysis of the Y-chromosome STR haplotypes of the Lauricocha individuals and a large database of modern indigenous South American males (cf. SI Materials and Methods) revealed that the haplotypes of individuals 1, 2, and 6 are closely related to haplotypes found in populations living in the Peruvian highlands today (S5 Fig). In addition, we determined that individual 1 exhibits the nine repeat allele (9RA) in the D9S1120 marker (S3 Table) recently described as a private allele only found in Native American and western Beringian populations [46]. The discovery of 9RA in an 8000 year old individual strengthens the hypothesis of an ancient and shared Beringian ancestry of this allele [46]. This is also supported by the observation that all typed Lauricocha individuals carry the derived homozygous C230 allele for marker rs9282541, in the cholesterol transporter ABCA1 (ATP-binding cassette transporter A1) gene, which is unique to the Americas [47]. Additionally, individuals 1, 2, 6, and 9 exhibit ABO blood type O1, found in ~99% of contemporary Native South Americans (S3 Table).

## Conclusion

Lauricocha is 2500–3500 ^14^Cyr younger than other early archaeological sites in the high altitude Andes like Guitarrero Cave, Pachamachay and Telarmachay [3,6], but harbors a larger number of skeletal remains with a remarkable morphological diversity, and the oldest complete mitochondrial genome and nuclear data reported from South America so far. The range of the obtained radiocarbon dates from the Lauricocha individuals shows that they cannot be considered as a single biological population, a result relevant especially when reviewing older morphometric studies that used the skeletons found at this site as an example for Paleoamerican populations in western South America. This, and the small number of samples, limit our potential to perform standard population genetic analyses. Based on simple comparison, the combined results of all genetic markers studied here place the Lauricocha individuals within the expected genetic diversity observed in other pre-Columbian Central Andean populations (cf. [38,48]), and suggest that both pre-Columbian and modern Central Andeans trace parts of their ancestry back to the same small founding population. As it stands, the first results from early South Americans settlers support, rather than challenge, a single entry during the initial peopling of South America [42]. However, genome wide data from Lauricocha and other Early/Middle Holocene skeletons will be needed to elucidate the full peopling history of the Central Andes and of the Americas in general.

## Supporting Information

S1 Fig Calibrated radiocarbon dates including depth information. (TIF)

S2 Fig General anatomical overview of the Lauricocha skeletons. (TIF)

S3 Fig Skulls of individuals 2 and 8. (TIF)

S4 Fig Skull of individual 6. (TIF)

S5 Fig Y-chromosomal haplotype networks. (TIF)

S1 Table Radiocarbon dates and calibrations. (DOCX)

S2 Table PCR primer sequences. (DOCX)

S3 Table Nuclear and mitochondrial PCR based genotyping results. (XLSX)

S4 Table Sequencing statistics and mitogenome SNP calls. (XLSX)

S5 Table PCR and genotyping results for autosomal markers. (XLSX)

S1 Text Supplementary Material and Methods. (DOCX)
