# Supplementary figures and images for: A Re-Appraisal of the Early Andean Human Remains from Lauricocha in Peru
Source: PLoS One. 2015 Jun 10;10(6):e0127141. doi: 10.1371/journal.pone.0127141 (PMC4464891; doi:10.1371/journal.pone.0127141)

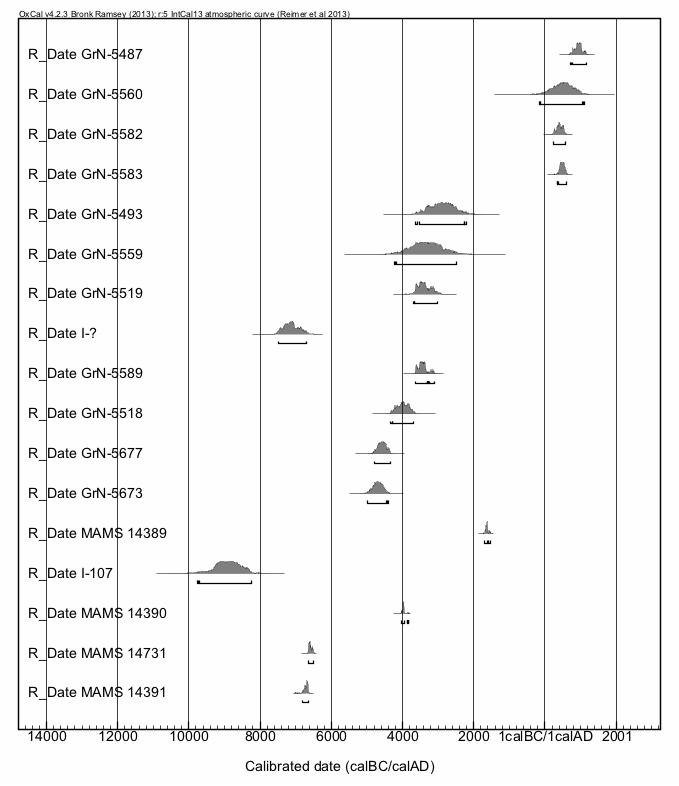

Supplement: S1 Fig — (TIF) [file pone.0127141.s001.tif]

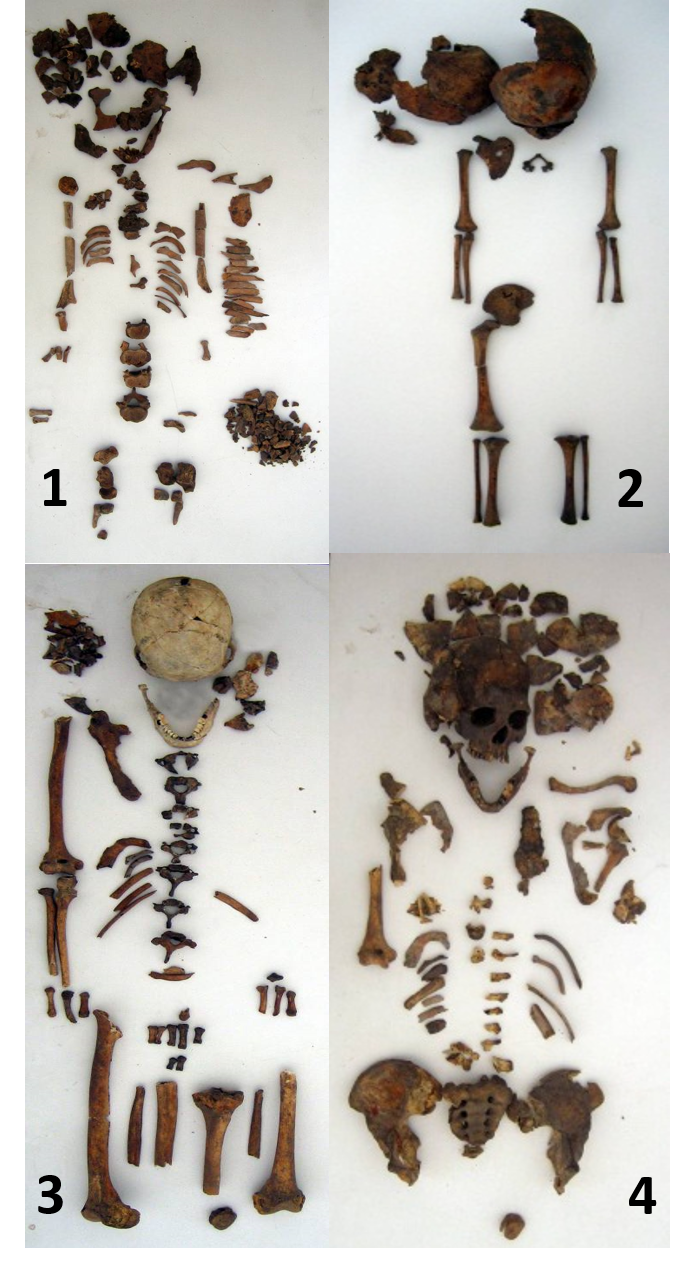

Supplement: S2 Fig — (TIF) [file pone.0127141.s002.tif]

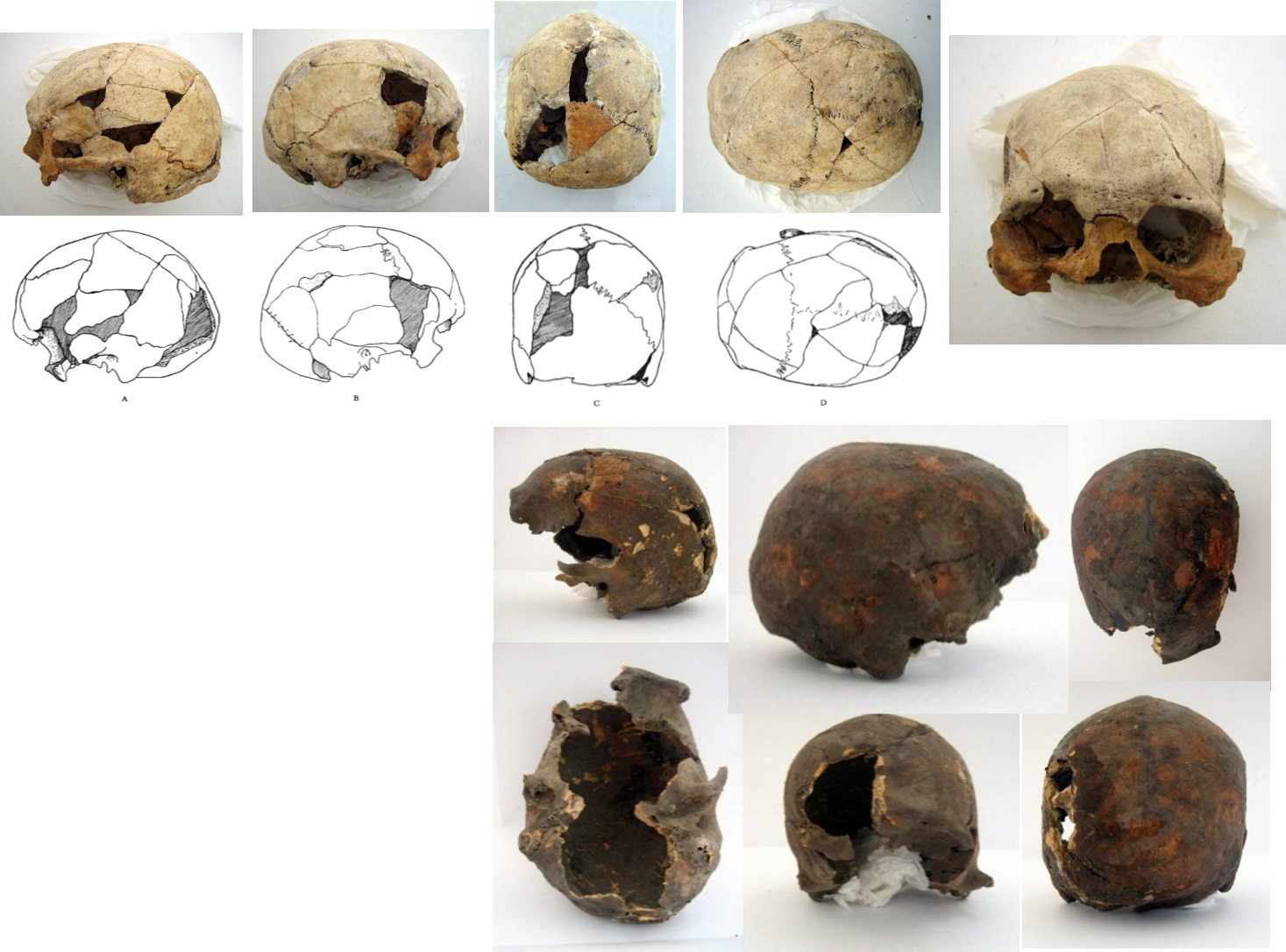

Supplement: S3 Fig — (TIF) [file pone.0127141.s003.tif]

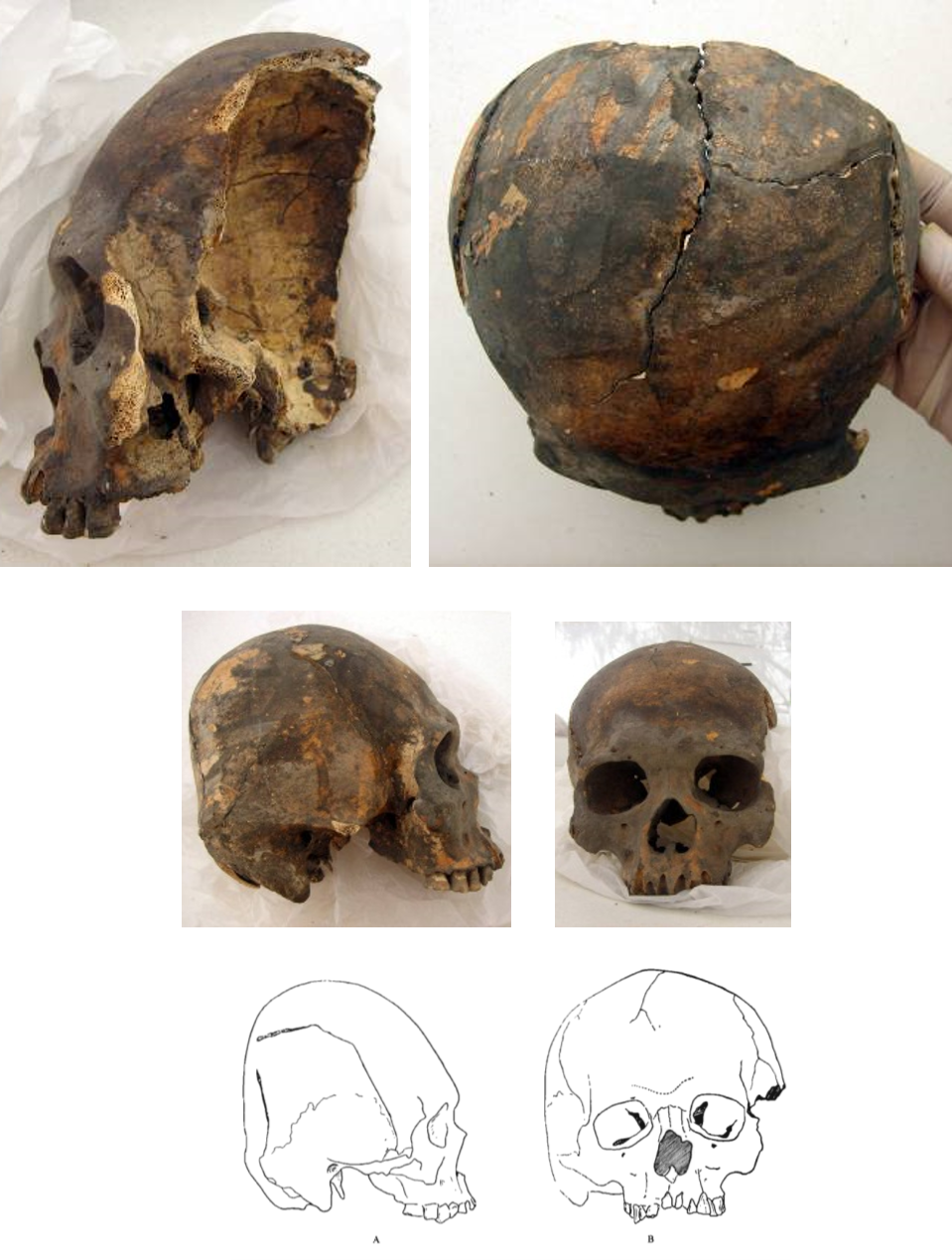

Supplement: S4 Fig — (TIF) [file pone.0127141.s004.tif]

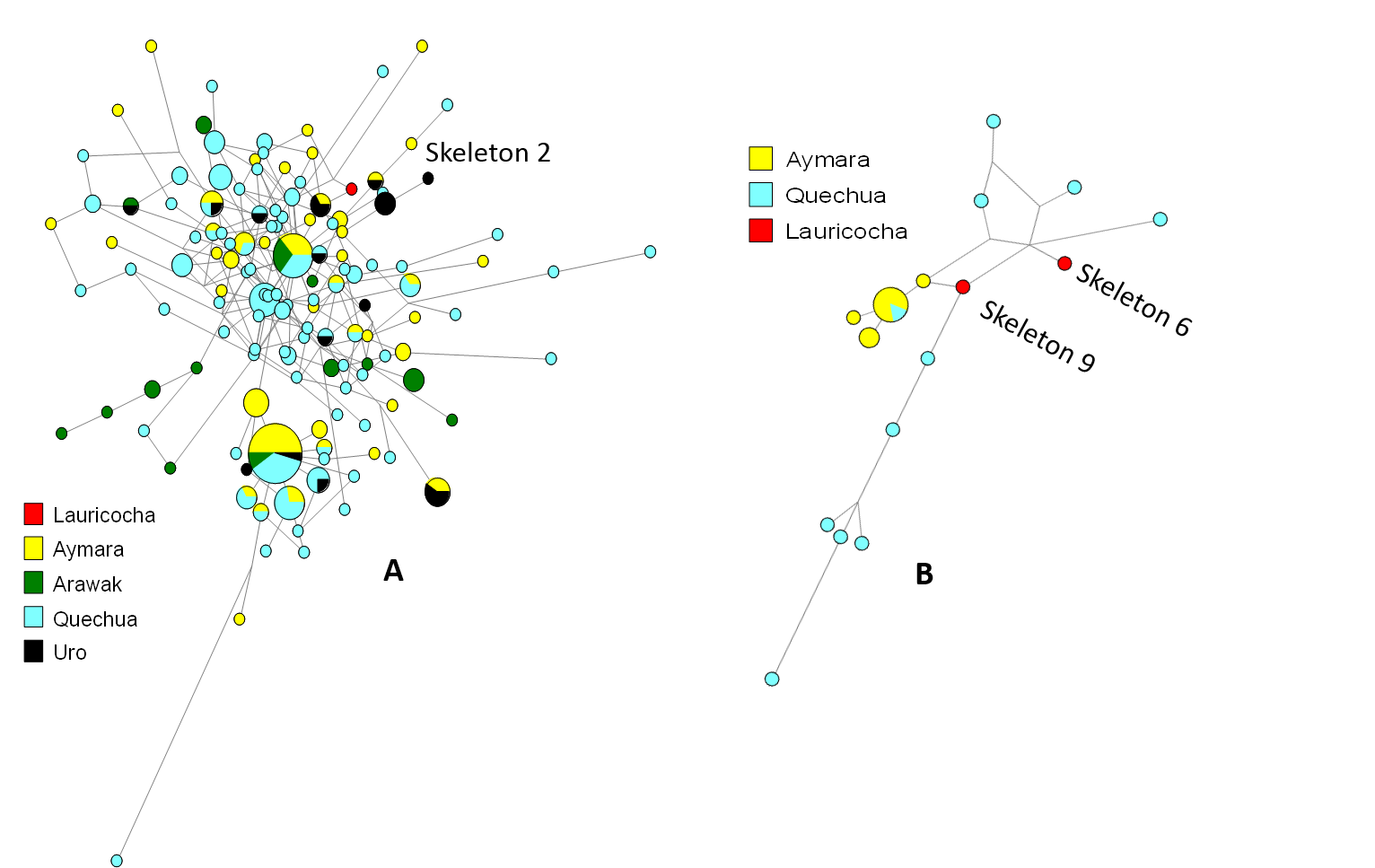

Supplement: S5 Fig — (TIF) [file pone.0127141.s005.tif]
