# Supplementary material for: A Re-Appraisal of the Early Andean Human Remains from Lauricocha in Peru
Source: PLoS One. 2015 Jun 10;10(6):e0127141. doi: 10.1371/journal.pone.0127141 (PMC4464891; doi:10.1371/journal.pone.0127141)
Supplement: S1 Table — (DOCX) [file pone.0127141.s006.docx]

S1 Table. Data of the Radiocarbon samples, calibrated with Intcal13.

| **Labcode** | **Depth (cm)** | **Info** | **Remarks** | **C14age** | | **cal Age min** | **cal age max** | **cal Age min** | **cal age max** | **δ13C** | **C-content [%]** | **C/N ratio** | **% Collagen** | **average F14C** | |
| --- | --- | --- | --- | --- | --- | --- | --- | --- | --- | --- | --- | --- | --- | --- | --- |
|  |  |  |  |  | +/- | **1 sigma** | **1 sigma** | **2 sigma** | **2 sigma** |  |  |  |  |  | +/- |
| ***GrN-5487*** | 90 | Cave L-2 Layer 12 res | Associated with ceramics | 1080 | 90 | 778 | 1032 | 718 | 1157 | -24,2 |  |  |  |  |  |
| ***GrN-5560*** | 90 | Cave L-2 Layer 12 humic | Associated with ceramics | 1500 | 280 | 223 | 862 | -160 | 1120 | -24,4 |  |  |  |  |  |
| ***GrN-5582*** | 120 | Cave L-2 Layer 14 Sed | Associated with ceramics | 1640 | 70 | 336 | 535 | 244 | 565 | -23,4 |  |  |  |  |  |
| ***GrN-5583*** | 150 | Cave L-2 Layer 18 sed | Associated with ceramics | 1570 | 60 | 421 | 547 | 354 | 613 | -23,4 |  |  |  |  |  |
| ***GrN-5493*** | 225 | Cave L-2 Layer 24 res | Lauricocha II | 4260 | 250 | -3328 | -2500 | -3627 | -2206 | 23,3 |  |  |  |  |  |
| ***GrN-5559*** | 225 | Cave L-2 Layer 24 humic | Lauricocha II | 4620 | 350 | -3761 | -2898 | -4230 | -2473 | -23,5 |  |  |  |  |  |
| ***GrN-5519*** | 275 | Cave L-2 Layer 28 res | Assoc. Lauricocha II | 4650 | 130 | -3635 | -3127 | -3695 | -3018 | -23,8 |  |  |  |  |  |
| ***I-?*** | ~305 | Cave L-2 Layer P Lauricocha II | Assoc. Lauricocha II | 8140 | 140 | -7445 | -6838 | -7486 | -6700 |  |  |  |  |  |  |
| ***GrN-5589*** | 305 | Cave L-2 Layer 31 humic | Assoc. Lauricocha II | 4660 | 90 | -3629 | -3354 | -3641 | -3106 | -23,5 |  |  |  |  |  |
| ***GrN-5518*** | 305 | Cave L-2 Layer 31 res | Assoc. Lauricocha II | 5170 | 140 | -4226 | -3797 | -4326 | -3696 | -24 |  |  |  |  |  |
| ***GrN-5677*** | 310 | Cave L-2 Layer 32 humic |  | 5720 | 110 | -4701 | -4457 | -4798 | -4349 | -23,7 |  |  |  |  |  |
| ***GrN-5673*** | 310 | Cave L-2 Layer 32 res |  | 5830 | 120 | -4832 | -4592 | -4997 | -4403 | -23,9 |  |  |  |  |  |
| ***MAMS 14389*** | 310 | Lau3 -Skeleton 6 | Metatarsal | 3340 | 22 | -1682 | -1611 | -1690 | -1534 | -22 | 41,7 | 3,4 | 2,8 | 0,66 | 0,0018 |
| ***I-107*** | ~ 315 | Layer R | burnt & unburnt bone & charcoal | 9525 | 260 | -9253 | -8559 | -9755 | -8249 |  |  |  |  |  |  |
| ***MAMS 14390*** | 315 | Lau4 -Skeleton 2 | Metatarsal | 5160 | 27 | -3988 | -3955 | -4041 | -3825 | -27 | 39,1 | 3,4 | 4,2 | 0,5261 | 0,0017 |
| ***MAMS 14731*** | 330 | Lau5 - Skeleton 1 | Metatarsal I, right | 7760 | 31 | -6641 | -6534 | -6646 | -6504 | -27 | 39,1 | 3,5 | 1,3 | 0,3753 | 0,0014 |
| ***MAMS 14391*** | 340 | Lau1, Sekleton 9 | Pars petrosa | 7870 | 30 | -6750 | -6648 | -6821 | -6640 | -16 | 25,7 | 3,4 | 6,6 | 0,3808 | 0,0015 |
| ***GrN-5515*** | 90 | Layer 12 carbonates | Associated with ceramics | 24141 | 400 |  |  |  |  | 2,1 |  |  |  |  |  |
| ***GrN-5516*** | 225 | Layer 24 carbonates | Assoc. Lauricocha II | 28391 | 440 |  |  |  |  | 1,8 |  |  |  |  |  |
