# Supplementary material for: A Re-Appraisal of the Early Andean Human Remains from Lauricocha in Peru
Source: PLoS One. 2015 Jun 10;10(6):e0127141. doi: 10.1371/journal.pone.0127141 (PMC4464891; doi:10.1371/journal.pone.0127141)
Supplement: S2 Table — (DOCX) [file pone.0127141.s007.docx]

S2 Table. Previously unpublished PCR primers employed in this study (decaPlex autosomal miniSTR assay and mitochondrial long range PCR)

| Marker | Primer | Fluorescence Dye (5‘) | Primersequence (5‘ → 3‘) | Allele | Fragment-length-range [bp] | final conc. in PCR |
| --- | --- | --- | --- | --- | --- | --- |
| **Amelo-genin** | Up  low | 6FAM | CCTGGGCTCTGTAAAGAATAGTG  AGCTGATGGTAGGAACTGTAAAAT | X  Y | 86  92 | 0,06  0,06 |
| **D9S1120** | Up  low | 6FAM | TGTGTGGGTATATATTATATGTAGTGTATAGA  ATTCTTACTATAGAACATAGGACTCACATATT | 9 – 19 | 102 – 142 | 0.16  0.16 |
| **VWA** | Up  low | 6FAM | TGATAAATAGATACATAGGTTAGATAGAGATAG  ACTAGTGGATGATAAGAATAATCAGTATGT | 10 – 24 | 145 – 201 | 0.16  0.16 |
| **D16S539** | Up  low | HEX | CTCTTCCCTAGATCAATACAGACAG  ACATCTCTGTTTTGTCTTTCAATGA | 4 – 16 | 77 – 125 | 0.20  0.20 |
| **D7S820** | Up  low | HEX | ACCAAATATTGGTAATTAAATGTTTACTA  GGGTATGATAGAACACTTGTCATAGTT | 5 – 16 | 130 – 174 | 0.20  0.20 |
| **D21S11** | Up  low2 | HEX | CCAAGTGAATTGCCTTCTATCTA  GTTGTATTAGTCAATGTTCTCCAGAG | 24 – 38 | 178 – 234 | 0.20  0.20 |
| **D3S1358** | Up  low | NED | TCTCTTATACTCATGAAATCAACAGAG  GAGCAAGACCCTGTCTCATAGA | 9 – 20 | 92 – 136 | 0.20  0.20 |
| **D2S1338** | Up  low2 | NED | GCCCATAATCATGAGTTATTCAGT  ACATAATCCAGCTGTGGGAGG | 10 – 28 | 139 – 211 | 0.20  0.20 |
| **D19S433** | Up  low3 | ROX | ATAAAAATCTTCTCTCTTTCTTCCTCT  AGAATAAGATTCTGTTGAAGGAAA | 5.2 – 18.2 | 75 – 127 | 0.30  0.30 |
| **D8S1179** | Up  low | ROX | ACGGCCTGGCAACTTATATG  ATTGCGTGAATATGCCTTAATTTA | 7 – 19 | 137 – 185 | 0.30  0.30 |
| **Primer Name** |  |  | **Primer sequence (5’-3’)** |  | **Expected length (bp)** |  |
| **H2698-tailT7** |  | 5’-AATTGTAATACGACTCACTATAGGGTTGACCTGCCCGTGAAGAGG-3’ | | | 5721 |  |
| **L8351** |  | 5’-TTGGGGCATTTCACTGTAAAGAGG-3’ | | |  |  |
| **H7801-tailT7** |  | 5’-AATTGTAATACGACTCACTATAGGGCTATCCTGCCCGCCATCATC-3’ | | | 6530 |  |
| **L14267** |  | 5’-GAGGGGTCAGGGTTGATTCG-3’ | | |  |  |
| **H13305** |  | 5’-TCGGCATCAACCAACCACAC-3’ | | | 6735 |  |
| **L3407-tailT7** |  | 5’-AATTGTAATACGACTCACTATAGGGTACAACGTTGGGGCCTTTGC-3’ | | |  |  |
