# Supplementary material for: A Re-Appraisal of the Early Andean Human Remains from Lauricocha in Peru
Source: PLoS One. 2015 Jun 10;10(6):e0127141. doi: 10.1371/journal.pone.0127141 (PMC4464891; doi:10.1371/journal.pone.0127141)
Supplement: S1 Text — (DOCX) [file pone.0127141.s011.docx]

**Supplementary Text 1: Materials and Methods**

**Human Osteology**

The skeletons are in a very incomplete state, fragmented, fragile, and some of the skulls show post mortem plastic deformation (S2 Fig.). Apparently, during excavation or first analysis some consolidate was applied to stabilize the bones so that earth was adhered and also some bones were glued together. Also, the adhesive that was used to reconstruct the skulls is now degraded and looks transparent, yellowish and brittle. Some fragments that were pasted with this glue are now detached. This general state of preservation had inhibiting impact on the morphological inspections. Besides, the skull of individual 2 (S3 Fig.) was exhibited in the “Galería Orígenes” of MNAAHP for a long time, and the exposition to UV radiation discolored it considerably.

Some skeletons were found partially mixed with other individuals. We identified and separated the commingled bones with the help of old labels on the bones, when present. If no labels were found we used similarities in age, size, color etc. and compared the resulting re-assembled skeletons with the inventory of Bormida [1]. In some cases there were no enough information and some bones remained commingled. When comparing the existing bones with the inventories of Bormida in some cases we identified more bones and in some cases we didn’t find all the bones mentioned by him. Also we found many animal bones, some showing evidence of heating. The discrepancies between our inventory and Bormida’s can be explained in part because in the first half of the 20th century physical anthropologists were mainly concerned with the study of skulls and had little experience with post-cranial elements.

The sex of adults was estimated using cranial traits [2], as only individual 6 had a partially preserved pelvis. In all instances the sex estimated with morphological traits coincided with the genetic sex determination. To estimate the age of the only child (individual 9) of the sample we used the development of teeth [3] and the maturation of secondary centers of ossification [4]. The only bones available for adult age estimation were the first ribs and the teeth, so the methods of DiGangi et al. [5] and Ubelaker and Parra [6] were used. Stature was calculated only for individual 6, using the formula of Genovés [7]. Teeth were recorded using the recommendations of Buikstra and Ubelaker [2] and Hillson [8], with the modifications proposed by Tomasto-Cagigao [9]. Pathologies were observed with the help of a 10x lens and were described following Ortner [10]. Also some general observations of muscular attachments and indicators of activity were done using Hawkey and Merbs [11] and Capasso et al [12].

Skeletal remains of individual 1 could be identified as those of an elderly woman, skeleton 2 was a young male, approximately 25-years-old, skeleton 6 was a ~40-year-old male, skull 8 belonged to an adult male, and skeleton 9 was a 2-4-years-old child. No skeleton had complete long bones to calculate stature. Bormida reports a measurement of 421 mm. for the right femur of individual 6. Using this measurement we calculated a stature of 1.58 m – 1.65 m. which is comparable to average statures calculated for later Andean populations [9,13–15].

None of the skulls presented clear evidences of intentional cranial modification. In skull 2 the area around Lambda is slightly flattened, but it doesn’t seem to be the result of a deforming device. In skeleton 6, even though the frontal bone is not round, it does not show marks of pads or other elements used in deforming devices (S4 Fig.). Moreover, at first glance the occipital bone and posterior part of parietals seem to be flattened, but more than a half of the occipital is absent, and the parietals have a transversal crack in the posterior third that modifies their orientation causing the false impression of flattening.

**Ancient DNA Analysis**

We collected eight samples from the five Lauricocha individuals at the MNAAHP taking all possible precautions through the use of gloves, face masks, caps, long-sleeved clothes and decontamination of the working surface and tools with full strength commercial bleach. For individuals 1, 2, and 6 we collected each two samples: one molar, and one metatarsal or metacarpal. For individuals 8 and 9 we sampled complete petrosal bones. Teeth were extracted from alveolar sockets and petrosal bone was cut from the temporal using a Dremel® drill with diamond disk. The samples were initially shipped to the ancient DNA (aDNA) facilities at the Department of Anthropology, University Goettingen, Germany (GoA) where they were stored at -20°C. Hereafter, genetic investigations were conducted independently at three additional ancient DNA laboratories: the Australian Centre for Ancient DNA, University of Adelaide, Australia (ACAD), the Reich Lab at the Department of Genetics, Harvard Medical School, USA (HMS), and the Human Palaeogenomics lab at the Department of Anthropology, UCSC, Santa Cruz, USA (UC-HPL). Bone powder samples were distributed to these labs from GoA.

**Sample Preparation, DNA Extraction, PCR Amplification, and Analysis (GoA, UC-HPL)**

All pre-PCR analyses were carried out in laboratories entirely dedicated to ancient DNA analysis. All analyses were carried out according to the precautions and contamination prevention strategies described in refs. [16–18].

Upon arrival at GoA ~2g pieces from the bone samples were cut and removed using a Dremel® drill and sent to MAMS for AMS radiocarbon dating. The remaining samples (bone or teeth) were decontaminated upon entry to the aDNA laboratory by immersion in 6% sodium hypochlorite solution (bleach) for 10 minutes, afterwards rinsed with sterile ddH2O, and dried in a UV cross-linker for 1h. Parts of the specimen to be further processed were removed using a Dremel® drill. Samples were then pulverized using a Mixer Mill (MM400, Retsch).

At GoA two aDNA extractions were performed for all samples. At least two extraction blank controls (EBCs) were used in every batch of extractions. For each extract 0.1g of bone / tooth powder was digested and decalcified in extraction buffer consisting of 4 mL 0.5 M EDTA (pH 8.0) and 50μL Proteinase K (conc. 20 mg/mL, Qiagen) for 18 hours at 37°C under permanent inversion. Subsequently another 50μL Proteinase K (Invitrogen) was added and incubation continued for 2 hours at 56°C. The lysate was then mixed with 20 mL Binding Buffer (PB, Qiagen) and 1.6mL 3M sodium acetate pH 5.2 (Sigma-Aldrich) and run through a silicate membrane column (MinElute, Qiagen) with attached 10ml extender (Qiagen) employed on a vacuum manifold (QiaVac24, Qiagen) for DNA isolation and purification. The following washing step was performed using PE Buffer (Qiagen), following the recommendations of the manufacturer. Washing was repeated three times. DNA was then eluted from the column membranes with 60μL 56°C warm TTE buffer. The DNA extracts were directly stored at -20°C. Mitochondrial haplogroups and haplotypes for the samples were determined at GoA and ACAD by employing a 26-plex Single Base Extension (SBE) assay and the amplification of the HVR1 region of the mitochondrial genome using four pairs of overlapping primers as described in ref. [19,20]. Both laboratories generated concordant results.

All PCRs amplifying nuclear DNA targets were performed at GoA and UC-HGL. To ensure authenticity of the amplification results and to prevent false genotype calls due to allelic dropout we at least performed four independent amplifications per DNA extract for each genetic marker analyzed. For each individual we generated at least two independent DNA extracts resulting in a minimum of eight amplification results per marker used to call a consensus genotype. Alleles were only called if they were typed in over 50% of the amplifications. To further test if the DNA extracted was degraded DNA and not a modern contamination we performed quantitative PCR (qPCR),amplifying a 125bp and a 199bp nuclear DNA (nDNA) target as described in ref. [18]. Both fragments were amplified using the same upper primer but differing lower primers. Both experiments were designed to have the exact same PCR efficiency. For all DNA extracts qPCR showed that DNA quantity increases with decreasing fragment length by a ratio of 1:5 as to be expected for ancient DNA [21]. Finally, to test for contamination we analyzed autosomal STR genotypes for each sample by amplifying 14 autosomal microsatellites in two PCR reactions with overlapping markers. Because of the highly individual character of the genetic profiles generated by this method it is used as a standard forensics to identify individuals and to test kinship between individuals. The highly discriminating STR genotypes (genetic fingerprints) were compared for each DNA extract of a sample to monitor authenticity, and between independent samples of an individual (for individuals 1, 2, and 6) to test whether bone and teeth derived from the same individual. These tests were consistent in all cases (S3 Table). For the Y-chromosomal SNPs, and autosomal SNPs first amplifications were performed at GoA and subsequent amplifications at UC-HGL with concordant results.

Fourteen autosomal microsatellites (D13S317, D21S11, D18S51, TH01, D5S818, FGA, D9S1120, VWA, D16S539, D7S820, D3S1358, D2S1338, D19S433, D8S1179) and the sex specific locus Amelogenin were amplified in 2 multiplex reactions. Amplification and downstream analysis for the first heptaplex MiniSTR assay were performed as described in ref. [18]. Primer design and PCR evaluation for the second, new, DecaPlex MiniSTR assay followed the same strict rules as described in ref. 12. Primer sequences are given in S2 Table. The accuracy of STR genotyping results for the self-developed miniSTR assay were verified through the extensive comparison of allele typing results with a validated commercial kit using modern DNA samples with known and confirmed genotypes from the GEDNAP (German DNA profiling group) blind trials 38/39 from 2009 [18,22]. PCR were performed in 25μL reactions containing: 12,5 μL QIAGEN Multiplex PCR Mastermix plus (Qiagen, Hilden), 1,98 μL primerset (concentrations in S2 Table), 8mM ammonium sulfate (Sigma-Aldrich), 2-5μL DNA-extract, and DNase free RT-PCR grade H_2_O (Ambion®, Austin, TX) to complete the final reaction volume. PCR amplification took place in a Mastercycler (Eppendorf, Hamburg, Germany) under the following conditions: initialization for 5 min at 95 °C, followed by 40 cycles of 1 min 94 °C, 1 min 56 °C, and 2.5 min 59 °C; final elongation at 60°C for 45 min. PCR success and PCR product quantity for all amplifications were checked by gel electrophoresis on 2.5% agarose gels. Fragment lengths of the STRs were subsequently analyzed on an ABI Prism 310 genetic Analyzer (Applied Biosystems) using POP4. Both PCRs were performed at least four times per DNA extract and consensus genotypes were build using the independent PCR results for each sample (S3 Table).

ABO blood types were determined using a multiplex SBE PCR co-amplifying 4 SNPs that allow determination of five common ABO alleles (A, B, 01, 01V, and 02). PCRs, downstream analysis, and genotype / phenotype calling were performed as described in ref. [22]. ABO typing results can be found in S3 Table.

The rs9282541 SNP in the cholesterol transporter ABCA1 (ATP-binding cassette transporter A1) gene [23] was determined by PCR. Primers for the marker were designed using the Primer Select software (Lasergene 8.0 package, DNAstar) following the recommendations described in ref. [24]. Primer sequences are given in S2 Table. PCR was carried out in a total volume of 25μL containing 12.5 μL Qiagen Multiplex PCR Master Mix Plus (Qiagen, Hilden, Germany), 0.24 μM concentration for each of the primers, 1–4 μL of DNA template, and DNase free RT-PCR grade H_2_O (Ambion®, Austin, TX) to complete the final reaction volume. PCR amplification took place in a Mastercycler (Eppendorf, Hamburg, Germany) under the following conditions: initialization at 95°C for 5 min; 40 cycles at 94°C for 1 min, 59°C for 2.5 min; final elongation at 60°C for 30 min. PCR success and PCR product quantity were checked by gel electrophoresis on 2.5% agarose gels. Subsequently, PCR products were purified using MinElute columns (Qiagen) following the recommendations of the manufacturer. PCR products were then analyzed by direct sequencing of the heavy and light strands on an ABI Prism 310 Genetic Analyzer (Applied Biosystems) using POP6. Genotyping results for both SNPs can be found in S3 Table.

The Y-chromosomal haplogroup for the male samples was determined employing a multiplex SBE assay allowing the synchronous amplification and analysis of eight SRY SNPs (M3, M194, M130, M199, M19, M242, MEH2, M346; ISOGG Tree v. 8.89). Amplification, downstream analysis, and SNP calling was performed as described in [17]. SNP calls are listed in S3 Table. In addition, we determined the minimal Y-chromosomal haplotype for the male individuals employing a multiplex PCR amplifying eight Y-chromosomal STRs (DYS 392, DYS 391, DYS 19, DYS 389I, DYS 390, DYS 389II, DYS 393, DYS 385) as described in ref. [25]. Each PCR was performed at least two times per DNA extract. Consensus genotypes are given in S3 Table. We compared the Y-chromosomal haplotypes of the Lauricocha individuals to 363 Q-M3 (Q1a3a1) and 24 Q-L54* (Q1a3a1*) haplotypes of male modern South American individuals published by Sandoval et al. [26]. Haplotype network analyses were performed for both haplogroups using the Network software version 4.612 [27] employing parameters described in ref. [26]. The resulting networks are shown in S5 Figure.

The genotyping results of each individual PCR amplifying nuclear genetic markers can be found in S5 Table.

**DNA Extraction, library preparation, mitochondrial capture (ACAD)**

Bone powder from individuals 1, 2, 6, and 9 was sent to the Australian Centre for Ancient DNA for processing. DNA extraction was performed by slightly modifying the protocol from Brotherton et al. [28]. For each individual, 200 mg of bone powder were incubated overnight under constant rotation at 37°C in 4 mL of 0.5M EDTA, pH 8.0. The next day, lysis was achieved by adding 1.2 mg of proteinase K and incubating for 2 hours under constant rotation at 55°C. After lysis, samples were centrifuged at 4,600 rpm for 1min and the supernatant transferred to a 50 mL tube containing 125 µL of medium-sized silica suspension [28] and the following modified binding buffer: 13.5 mL QG buffer (Qiagen), 2.86 mL of 1X Triton, 20 mM NaCl, 0.2 M ammonium acetate (all Sigma-Aldrich). DNA was left to bind to silica for 1 hour at room temperature under constant rotation. The silica particles were pelleted by centrifuging at 4,600 rpm for 1 min and the supernatant was discarded. The pellet was transferred to a 1.5 mL tube and washed three times by resuspension in 1 mL 80% ethanol, centrifuged for 1min at 13,000 rpm and the supernatant discarded. The pellet was left to dry for 30 min and subsequently resuspended in 200 μL of pre-warmed (to 50°C) TE buffer (10mM Tris, 1mM EDTA) and incubated for 10 min. After pelleting for 1 min at 13,000 rpm the supernatant was collected, aliquoted and stored at -18°C until further use.

We built double-stranded Illumina libraries from 20 µL of each DNA extract following the protocol from Meyer & Kircher [29], using truncated Illumina adapters with dual 5-mer internal barcodes.

Mitochondrial RNA baits for DNA capture by hybridization were prepared in-house. In a first step, the whole mitochondrial genome of BL (hg H3k1) was amplified in three overlapping fragments using the Expand Long Range dNTPack kit (Roche), and following the manufacturer’s protocol with an annealing temperature of 60ºC and the primers in S2 Table. From this step onward, all commercial kits were used according to the manufacturer’s protocols. For each amplicon, one of the primers was 5’-tailed with a T7 promoter sequence (5’- AATTGTAATACGACTCACTATAGGG-3’) in order to perform an *in vitro* transcription using the T7 High Yield RNA Synthesis Kit (New England BioLabs). DNA was degraded with DNase after the *in vitro* transcription was completed. Resulting RNA was purified using the Ambion MEGAclear kit (Life Technology), and eluted in 2 x 50 µL of RNase-free molecular grade water. RNA was quantified using a Nanodrop spectrophotometer (FisherScientific), and RNA integrity was assessed using a TapeStation (Agilent). For each mitochondrial fragment, 40 µg of RNA was then fragmented using the NEBNext Magnesium RNA Fragmentation Module (New England BioLabs). Fragmented RNA was purified using the RNeasy Minelute Cleanup kit (Qiagen), and eluted in 15 µL of RNase-free molecular grade water. RNA was quantified using a Nanodrop spectrophotometer, and RNA fragmentation was assessed using a TapeStation. Finally, fragmented RNA was randomly biotynilated using the EZ-Link Psoralen-PEG3-Biotin kit (Thermo Scientific), performing as many assays as was possible given the concentration of starting template. Biotynilated RNA was purified using the RNeasy Minelute Cleanup kit (Qiagen), and eluted in 15 µL of RNase-free molecular grade water. Biotynilated RNA baits from the initial three mitochondrial fragments were pooled at a concentration of 50 ng/µL each (150 ng/µL total) for capture by hybridisation.

For the capture assay, 200-250 ng of barcoded DNA library were mixed with 2.5 µg of salmon sperm DNA and 2.5 µg of Cot1 DNA in a volume of 8 µL. We prepared a RNA mix consisting of 800 ng of biotinylated RNA baits, 25 pmole of blocking RNA oligonucleotides (matching the sequence of the truncated Illumina P5 and P7 adapters), and 20 U of SUPERase-In (Ambion) in a volume of 6.5 µL. The hybridisation buffer contained 5X SSPE, 10 mM EDTA pH=8.0, 10X Denhart’s solution, and 0.2% SDS in a volume of 18.4 µL. The DNA mix was denaturated at 94ºC for 5 min. Hybridisation buffer, RNA and DNA were incubated at 65ºC for 5 min separately, then mixed and incubated as follows: 65ºC for 9h, 63ºC for 9h, 61ºC for 9h, 59ºC for 9h, 57ºC for 9h, 60ºC for 3h.

For each hybridisation reaction, 50 µL of Dynabeads® MyOne Streptavidin C1 (Life Technologies) were washed two times with 500 µL of 2X SSC + 0.05% Tween-20 at room temperature. Beads were then incubated with 100 µg of yeast tRNA in 500 µL of 2X SSC + 0.05% Tween-20 for 30 min under constant rotation at room temperature, to saturate all sites on the beads that bind nucleic acids in a non-specific manner. Beads were then washed with 500 µL of 2X SSC + 0.05% Tween-20 at room temperature and resuspended in 200 µL of 2X SSC + 0.05% Tween-20. The hybridisation reaction was incubated with the beads for 30 min under constant rotation at room temperature to allow binding of the biotin to the streptavidin. A series of increasingly stringent washes was then performed: one wash with 500 µL of 2X SSC + 0.05% Tween-20 for 10 min at room temperature, two washes with 500 µL of 0.75X SSC + 0.05% Tween-20 for 10 min at 60ºC, and one washes with 500 µL of 0.2X SSC + 0.05% Tween-20 for 10 min at 60ºC. After the last wash, beads were directly used for off-bead PCR amplification, using full-length indexed Illumina adapters as described in Meyer & Kircher [29].

We pooled indexed libraries with other libraries and sequenced them on a lane of Illumina HiSeq2000 with the v3 chemistry, using 2x100 cycles, at the Australian Cancer Research Foundation (ACRF) Cancer Genomics Facility, Adelaide, South Australia. Reads were automatically demultiplexed by index using Casava and a strict matching of the 7-mer indexes. We performed an additional demultiplexing based on a strict matching of the two 5-mer internal barcodes and we trimmed the barcodes using the program Sabre 1.0 (https://github.com/najoshi/sabre). We used AdapterRemoval v1.5.2 [30] with default parameters to trim residual adapter sequences and merge overlapping reads. Merged reads were aligned to the Reconstructed Sapiens Reference Sequence (RSRS) sequence [31] with bwa 0.7.5a-r405 [32], using the parameters space recommended for ancient DNA (no seed, one gap opening, relaxed edit distance) [33]. Duplicate reads were removed using FilterUniqueSAMCons.py [34] and final pileup statistics were calculated using SAMtools. Reads pileups were visualized in Geneious v7.1.3 (Biomatters. Available from http://www.geneious.com/). SNPs were called in Geneious for all polymorphisms with minimum coverage 2 and a minimum variant frequency 0.7. The assembly and the resulting list of SNPs were verified by eye and compared to SNPs reported at phylotree.org (mtDNA tree Build 16 [19 Feb 2014]) [31]. Following recommendations in van Oven et al. 2009 [35] we excluded common indels at nucleotide positions 309.1C(C), 315.1C, AC indels at 515-522, 16182C, 16183C, 16193.1C(C), and C16519T. Haplotype calls are given in S4 Table.

**DNA Extraction, library preparation, mitochondrial capture (HMS)**

A bone powder aliquot of individual 8 that only produced mitochondrial DNA results using PCR based methods was sent to the Reich lab at Harvard Medical School (HMS), Boston. 75 mg of the bone powder were used to extract DNA according to Dabney et al. [36]. Final elution was performed twice in 16-30 μL 1xTE buffer (with 0.05% Tween-20). 30 μL of the DNA extract were subsequently used to build a double-stranded Illumina sequencing library following the UDG- protocol described in Rohland et al. 2014 [37] employing adapters with internal barcodes. The library of individual 8 built at HMS and libraries for individuals 1, 2, 6, and 9 prepared at ACAD were then used for subsequent hybridization capture and sequencing of the mitochondrial genomes

Hybridization enrichment was performed as described elsewhere [38] with 239 baits targeting the human mitochondrial genome (3bp tiling based on NC_001807 as in reference [39]) using a semi-automated protocol in a 96-well plate setup on an Evolution P3 (Perkin Elmer, Waltham, USA) for two consecutive rounds. For each hybridization reaction, we used 500ng of single stranded bait library together with 500ng DNA library. The oligonucleotide blockers used for each of the respective adapter lengths are specified in ref. [37]. All other parameters of the hybridization, capture and washing steps and amplifications can be found in the original paper [38]. Each sample was subsequently indexed prior to sequencing with a unique index (pair), using 7-mer index sequences as in Meyer and Kircher [29].

We pooled indexed libraries (shotgun and mtDNA-captured with different index combinations) with several other libraries and sequenced them on a MiSeq instrument using v2 chemistry with the standard sequencing primers provided in the cartridges. We sequenced 2x75 cycles with the standard Nextera sequencing protocol (or TruSeq HT protocol) that reads both indices. We used the automatic demultiplexing provided by Illumina BaseSpace (allowing up to 1 mismatch per index). We then trimmed adapters and merged R1 and R2 sequences, requiring an overlap of at least 15bp (allowing one mismatch) using SeqPrep [40] (modified to require more conservatively that quality scores in the merged regions use the best score rather than aggregating the two inferred reliabilities of the base call), prior to trimming the barcodes (if applicable) from both ends of the merged molecules. For each sample, we restricted to reads that had the expected indices. We then only analyzed sequences that matched the expected 7mer barcodes, allowing up to two mismatches. Using bwa 0.6.1-r104 [32] we performed alignments twice, i) to the human reference genome (hg19) which contains the rCRS mitochondrial genome, and to ii) the Reconstructed Sapiens Reference Sequence (RSRS) mitochondrial sequence [31]. We computed target coverage with BEDtools version 2.16.2 [41] and used MapDamage2.0 [42] to compute damage rates and fragmentation patterns. We report the median length of all unique sequences aligned to the mitochondrial genome for samples for which we prepared multiple libraries with different protocols.

We estimated mtDNA contamination using a Markov Chain Monte Carlo based estimator (SI

5 in [43]). More precisely, we built a consensus sequence using a minimum base quality of 30 and a minimum coverage of 5, stripping gaps and ignoring any spurious heterozygote positions. We realigned all reads to this consensus, and then used the resulting alignments for estimation of contamination (SI 5 in [43]), trimming the first and last 5 bases from every read to minimize errors due to ancient DNA degradation. Information on sequencing coverage, damage estimates, and contamination ratio are found in Supplementary Dataset 2.

**Phylogenetic analysis of the mitochondrial data**

We used the SNP calls reported in S4 Table to embed the newly typed Lauricocha samples in the existing mitochondrial phylogeny (mtDNA tree Build 16 [19 Feb 2014] available at www.phylotree.org) [35]. This was done manually by following the hierarchical structure of the tree up to the most derived SNP shared with an existing sequence haplotype. Additional SNPs were considered to be private mutations. All five Lauricocha sequences represent unique branches along the stem of Native American haplogroups A2 and B2.

Individuals 6 and 8 share a common back mutation SNP at np 16311 [44], but differ by two coding region mutations (G5585A, G9163A) in individual 8, which is ~5000 years older. Based on these two differences and since T16311C! is a known recurrent mutation, we considered the placement of both individuals on separate branches to be more parsimonious.

**Supplementary references**

1. Bormida M (1964) Los Esqueletos de la Cueva Lauricocha. Acta Praeh V/VI: 1–33.

2. Buikstra JE, Ubelaker DH (1994) Standards for data collection from human skeletal remains.

3. Ubelaker DH (1989) Human Skeletal Remains. Excavation, Analysis, Interpretation. Washington DC: Smithonian Institution Press.

4. Scheuer L, Black S, Cunningham C (2000) Developmental juvenile osteology. New York: Academic Press.

5. Digangi EA, Bethard JD, Kimmerle EH, Konigsberg LW (2009) A new method for estimating age-at-death from the first rib. Am J Phys Anthropol 138: 164–176.

6. Ubelaker DH, Parra RC (2008) Application of three dental methods of adult age estimation from intact single rooted teeth to a Peruvian sample. J Forensic Sci 53: 608–611.

7. Genovés S (1967) Proportionality of the long bones and their relation to stature among Mesoamericans. Am J Phys Anthropol 26: 67–77.

8. Hillson S (2001) Recording dental caries in archaeological human remains. Int J Osteoarchaeol 11: 249–289.

9. Tomasto-Cagigao E (2009) Talking Bones: Bioarchaeological Analyzis of Individuals from Palpa. In: Reindel M, Wagner G, editors. New Technologies for Archaeology: Multidisciplinary Investigations in Palpa and Nasca, Peru. Berlin-Heidelberg-New York: Springer. pp. 141–158.

10. Ortner DJ, Putschar WG (1981) Identifcation of Pathological Conditions in Human Skeletal Remains. Smithsonian: 95–100.

11. Hawkey DE, Merbs CF (1995) Activity-induced Musculos keletal Stress Markers (MSM ) and Subsistence Strategy Changes among Ancient Hudson Bay Eskimos. Int J Osteoarchaeol 5: 324 – 338.

12. Capasso L, Kennedy K, Wilczak C (1998) Atlas of Occupational Markers on Human Remains. Rome: Museo di Storia delle Scienze Biomediche.

13. Orefici G (1996) Nuevos enforques sobre la transicion Paracas-Nasca en Cahuachi (Peru). Andes 1 : 173–189.

14. Sutter R, Cortez R (2007) Analysis of Human Sk eletal Materials from the Site of Kasapata. In: Bauer BS, editor. Kasapata and the Archaic Period of the Cusco Valley. Los Angeles: The Cotsen Institute of Archaeology Press. pp. 78–110.

15. Verano JW (1997) Advances in the Paleopathology of Andean South America. J World Prehist 11: 237–268.

16. Fehren-Schmitz L, Reindel M, Tomasto-Cagigao E, Hummel S, Herrmann B (2010) Pre-Columbian Population Dynamics in Coastal Southern Peru: A Diachronic Investigation of mtDNA Patterns in the Palpa Region by Ancient DNA Analysis. Am J Phys Anthropol 141: 208–221.

17. Fehren-Schmitz L, Warnberg O, Reindel M, Seidenberg V, Tomasto-Cagigao E, et al. (2011) Diachronic investigations of mitochondrial and y-chromosomal genetic markers in pre-columbian andean highlanders from South peru. AnnHumGenet 75: 266–283.

18. Seidenberg V, Schilz F, Pfister D, Georges L, Fehren-Schmitz L, et al. (2012) A new miniSTR heptaplex system for genetic fingerprinting of ancient DNA from archaeological human bone. J Archaeol Sci 39: 3224–3229.

19. Coutinho A, Valverde G, Fehren-Schmitz L, Cooper A, Barreto Romero MI, et al. (2014) AmericaPlex26: A snapshot multiplex system for genotyping the main human mitochondrial founder lineages of the Americas. PLoS ONE 9(3): e93292.

20. Fehren-Schmitz L, Reindel M, Tomasto-Cagigao E, Hummel S, Herrmann B (2010) Pre-Columbian population dynamics in coastal southern Peru: A diachronic investigation of mtDNA patterns in the Palpa region by ancient DNA analysis. Am J Phys Anthropol 141: 208–221.

21. Kirsanow K, Burger J (2012) Ancient human DNA. Ann Anat 194: 121–132.

22. Georges L, Seidenberg V, Hummel S, Fehren-Schmitz L (2012) Molecular characterization of ABO blood group frequencies in pre-Columbian Peruvian highlanders. Am J Phys Anthropol. 149(2):242-9.

23. Acuña-Alonzo V, Flores-Dorantes T, Kruit JK, Villarreal-Molina T, Arellano-Campos O, et al. (2010) A functional ABCA1 gene variant is associated with low HDL-cholesterol levels and shows evidence of positive selection in Native Americans. Hum Mol Genet 19: 2877–2885.

24. Hummel S (2003) Ancient DNA Typing: Methods, Strategies and Applications. Berlin-Heidelberg-New York: Springer.

25. Grumbkow P v., Frommer S, Kootker LM, Davies GR, Mazanec J, et al. (2013) Kinship and mobility in 11th-century A.D. Gammertingen, Germany: An interdisciplinary approach. J Archaeol Sci 40: 3768–3776.

26. Sandoval JR, Lacerda DR, Jota MS a, Salazar-Granara A, Vieira PPR, et al. (2013) The genetic history of indigenous populations of the peruvian and bolivian altiplano: the legacy of the uros. PLoS One 8: e73006.

27. Herrnstadt C, Elson JL, Fahy E, Preston G, Turnbull DM, et al. (2002) Reduced-median-network analysis of complete mitochondrial DNA coding-region sequences for the major African, Asian, and European haplogroups. AmJHumGenet 70: 1152–1171.

28. Brotherton P, Haak W, Templeton J, Brandt G, Soubrier J, et al. (2013) Neolithic mitochondrial haplogroup H genomes and the genetic origins of Europeans. Nat Commun 4: 1764.

29. Meyer M, Kircher M (2010) Illumina sequencing library preparation for highly multiplexed target capture and sequencing. Cold Spring HarbProtoc 2010: db.

30. Lindgreen S (2012) AdapterRemoval: Easy Cleaning of Next Generation Sequencing Reads. BMC Res Notes 5: 337.

31. Behar DM, Van Oven M, Rosset S, Metspalu M, Loogväli EL, et al. (2012) A “copernican” reassessment of the human mitochondrial DNA tree from its root. Am J Hum Genet 90: 675–684.

32. Li H, Durbin R (2009) Fast and accurate short read alignment with Burrows-Wheeler transform. Bioinformatics 25: 1754–1760.

33. Schubert M, Ginolhac A, Lindgreen S, Thompson JF, AL-Rasheid KA, et al. (2012) Improving ancient DNA read mapping against modern reference genomes. BMC Genomics 13: 178.

34. Kircher M (2012) Analysis of high-throughput ancient DNA sequencing data. Methods Mol Biol 840: 197–228.

35. Van Oven M, Kayser M (2009) Updated comprehensive phylogenetic tree of global human mitochondrial DNA variation. HumMutat 30: E386–E394.

36. Dabney J, Knapp M, Glocke I, Gansauge M-T, Weihmann A, et al. (2013) Complete mitochondrial genome sequence of a Middle Pleistocene cave bear reconstructed from ultrashort DNA fragments. Proc Natl Acad Sci U S A 110: 15758–15763.

37. Rohland N, Harney E, Mallick S, Nordenfelt S, Reich D (2014) Partial UDG-treatment for screening of ancient DNA. Phil Trans R Soc B 370: 20130624.

38. Fu Q, Meyer M, Gao X, Stenzel U, Burbano HA, et al. (2013) DNA analysis of an early modern human from Tianyuan Cave, China. Proc Natl Acad Sci U S A 110: 2223–2227.

39. Meyer M, Fu Q, Aximu-Petri A, Glocke I, Nickel B, et al. (2014) A mitochondrial genome sequence of a hominin from Sima de los Huesos. Nature 505: 403–406.

40. John JS (2011) SeqPrep: https://github.com/jstjohn/SeqPrep..

41. Quinlan AR, Hall IM (2010) BEDTools: A flexible suite of utilities for comparing genomic features. Bioinformatics 26: 841–842.

42. Jónsson H, Ginolhac A, Schubert M, Johnson PLF, Orlando L (2013) MapDamage2.0: Fast approximate Bayesian estimates of ancient DNA damage parameters. Bioinformatics. Vol. 29. pp. 1682–1684.

43. Fu Q, Mittnik A, Johnson PLF, Bos K, Lari M, et al. (2013) A revised timescale for human evolution based on ancient mitochondrial genomes. Curr Biol 23: 553–559.

44. Torroni A, Achilli A, Macaulay V, Richards M, Bandelt HJ (2006) Harvesting the fruit of the human mtDNA tree. Trends Genet 22: 339–345.

**Supplementary figure legends**

**S1 Fig.** Calibrated radiocarbon dates including depth information (top to bottom). The carbonate samples dated at Groningen with ages above 20,000 years are not included.

**S2 Fig.** General anatomical overview of the Lauricocha skeletons (1: skeleton 1; 2: skeleton 9; 3: skeleton 2; 4: skeleton 6).

**S3 Fig.** Skulls of individuals 2 (A) and 8 (B) from all perspectives (drawings by Bormida 1964).

**S4 Fig.** Skull of individual 6 from all perspectives (drawings by Bormida 1964).

**S5 Fig.** (A) Y chromosome STR network for 363 Q-M3 chromosomes published elsewhere (Sandoval et al. 2013). The Lauricocha individual 2 haplotype (red dot) is only 1 mutation step away from three Aymaran individuals from the Peruvian and Bolivian Altiplano (Santa Rosa de Yanaque and Uro-Poopo). (B) Y chromosome STR network for 24 Q-L54* chromosomes published elsewhere (Sandoval et al. 2013). The haplotypes of individuals Lauricocha 1 and 6 haplotypes (red dots) are related to haplotypes found around the Titicaca Lake (Santa Rosa de Yanaque), and Cajamarca (northern Andes of Peru), respectively.
